# Development of Novel Lightweight Dual-Phase Al-Ti-Cr-Mn-V Medium-Entropy Alloys with High Strength and Ductility

**DOI:** 10.3390/e22010074

**Published:** 2020-01-06

**Authors:** Yu-Chin Liao, Po-Sung Chen, Chao-Hsiu Li, Pei-Hua Tsai, Jason S. C. Jang, Ker-Chang Hsieh, Chih-Yen Chen, Ping-Hung Lin, Jacob C. Huang, Hsin-Jay Wu, Yu-Chieh Lo, Chang-Wei Huang, I-Yu Tsao

**Affiliations:** 1 Department of Mechanical Engineering, National Central University, Taoyuan 320, Taiwan; llllurker@gmail.com; 2 Institute of Material Science and Engineering, National Central University, Taoyuan 320, Taiwan; thepacific999@gmail.com (P.-S.C.); csuizeal5867@gmail.com (C.-H.L.); peggyphtsai@gmail.com (P.-H.T.); evauseonly@gmail.com (I.-Y.T.); 3 Department of Materials and Optoelectronic Science, National Sun Yat-Sen University, Kaohsiung 804, Taiwan; khsieh@mail.nsysu.edu.tw (K.-C.H.); cychen@mail.nsysu.edu.tw (C.-Y.C.); linnantou12345@gmail.com (P.-H.L.); 4 Institute for Advanced Study, Department of Materials Science & Engineering, City University of Hong Kong, Kowloon, Hong Kong, China; 5 Department of Materials Science and Engineering, National Chiao Tung University, Hsinchu 300, Taiwan; ssky0211@nctu.edu.tw (H.-J.W.); eulerycl@gmail.com (Y.-C.L.); 6 Department of Civil Engineering, Chung Yuan Christian University, Taoyuan 320, Taiwan; cwhuang@cycu.edu.tw; 7 R&D Center for Membrane Technology, Chung Yuan Christian University, Taoyuan 320, Taiwan

**Keywords:** high-entropy alloy, medium-entropy alloy, lightweight alloy, mechanical property

## Abstract

A novel lightweight Al-Ti-Cr-Mn-V medium-entropy alloy (MEA) system was developed using a nonequiatiomic approach and alloys were produced through arc melting and drop casting. These alloys comprised a body-centered cubic (BCC) and face-centered cubic (FCC) dual phase with a density of approximately 4.5 g/cm^3^. However, the fraction of the BCC phase and morphology of the FCC phase can be controlled by incorporating other elements. The results of compression tests indicated that these Al-Ti-Cr-Mn-V alloys exhibited a prominent compression strength (~1940 MPa) and ductility (~30%). Moreover, homogenized samples maintained a high compression strength of 1900 MPa and similar ductility (30%). Due to the high specific compressive strength (0.433 GPa·g/cm^3^) and excellent combination of strength and ductility, the cast lightweight Al-Ti-Cr-Mn-V MEAs are a promising alloy system for application in transportation and energy industries.

## 1. Introduction

High-entropy alloys (HEAs), also known as multiprincipal element alloys, have received considerable attention from the scientific community because of the high mixing entropy of alloying elements. These new-class alloys exhibit unique properties such as the high-entropy effect, distorted lattices, sluggish diffusion, and cocktail effect [1,2]. Lightweight HEAs (LWHEAs) have also gained attention due to the demand from energy and transportation industries. However, the development of LWHEAs is challenging due to the limited availability of light, nontoxic, and inexpensive elements. 

Some of the equiatomic LWHEAs composed of light elements such as Al and Ti have been investigated recently [3]. However, Al and Ti tend to form stable intermetallic compounds with many other elements due to their highly negative values of heat of mixing. Consequently, these brittle intermetallic phases exert detrimental effects on the mechanical properties of these LWHEAs [4,5]. Moreover, Li- and Mg-containing HEAs exhibited complex microstructures with a mixture of various intermetallic compounds [6,7]. Therefore, a simple microstructure and phase stability are highly essential to achieve the desirable mechanical properties for future promising applications. 

In 2015, a nonequiatomic design concept was widely adopted and led to a breakthrough [8]. This concept not only provided flexibility of HEA design but also contrasted the existing HEA design concepts. Numerous alloy combinations were created and the microstructures of selected alloys revealed a single phase [9,10,11]. When there is one element with high atomic percentage and the other elements have equal atomic percentage, the calculated configurational entropies of the alloys were changed from high-entropy to medium-entropy areas [12]. Moreover, the literature has reported that some medium-entropy alloys (MEAs) composed of ternary and quaternary alloys exhibited a single phase and demonstrated more promising mechanical properties compared with HEAs [13,14,15,16]. 

In this study, nonequiatomic lightweight Al-Ti-Cr-Mn MEAs were first designed to achieve high strength and appropriate ductility. To reduce the alloy density, a high Al content and other light elements with similar atomic radius were chosen to attain the light weight (alloy density is defined as <5 g/cm^3^). Subsequently, one alloy composition with a more favorable mechanical performance among the quaternary Al-Ti-Cr-Mn alloys was further modified by adding V to enhance its mechanical properties and phase stability at a high temperature [17].

## 2. Materials and Methods

### 2.1. Materials

Commercial raw materials, Al, Ti, Mn, Cr, and V, with purity >99.9 wt.%, were used for preparing the alloy ingots through arc melting under an Ar gas atmosphere. Each ingot was remelted four times under the Ar gas atmosphere to ensure chemical homogeneity. The drop-casting process was used to fabricate alloys with a dimension of 30 mm × 30 mm × 5 mm. The melting points were measured within the operating temperature range (25–1500 °C) by using a high-temperature differential scanning calorimeter (Netsch STA449F3, HT-DSC) at a heating rate of 20 K/s under an air atmosphere. The Homogenization treatment was conducted in a quartz tube furnace at 600, 800, and 1000 °C in a high vacuum atmosphere (10^−5^ Pa) for 24 h and then air cooling was performed. Density was measured using the Archimedes method.

### 2.2. Microstructure Characterization

X-ray diffraction (XRD, Bruker D2, Karlsruhe, Germany) was employed to characterize the crystal structure of alloy ingots by using Cu Kα radiation. The microstructure and chemical composition were characterized using a scanning electron microscope (UHR FE-SEM, Hitachi SU8220, Mannheim, Germany) equipped with an energy dispersive X-ray spectrometer and electron backscatter diffraction (EBSD, Oxford AztechHKL, Channel 5, Wiesbaden, Germany). The kernel average misorientation (KAM) approach was selected for the quantitative evaluation of small local strain gradients by using Channel 5.

### 2.3. Mechanical Testing

The hardness of alloy samples was measured using a Vickers hardness tester (model: Mitutoyo, HM-221) with a load of 1 kg for 10 s. At least five readings were measured at random areas in the specimen. Compression tests were conducted at room temperature in a universal testing machine (Hung Ta, HT9102) under quasi-static loading at an initial strain rate of 1 × 10^−4^ s^−1^.

## 3. Results 

### 3.1. Density of the As-Cast Alloys

Although Cr and Mn are heavy elements (~7.2 g/cm^3^), the Al-Ti-Cr-Mn and Al-Ti-Cr-Mn-V alloys with high Al content can achieve a low density of approximately 4.5 g/cm^3^, which is lower than 5 g/cm^3^. The measured densities of the alloys were close to theoretical densities, which were estimated using the rule of mixtures, as presented in Table 1.

### 3.2. Microstructure and Mechanical Properties of Quarternary Al-Ti-Cr-Mn MEAs

**Figure 1:**
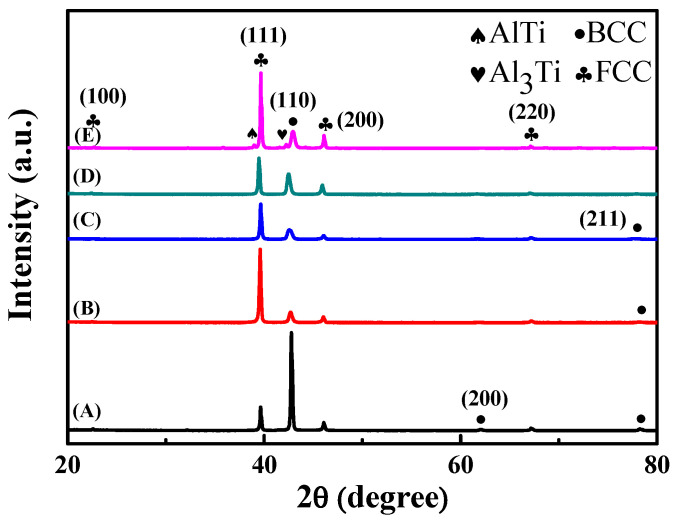
The XRD (X-ray diffraction) patterns of as-cast (**A**) Al_50_Ti_15_Cr_15_Mn_20_, (**B**) Al_50_Ti_20_Cr_10_Mn_20_, (**C**) Al_50_Ti_20_Cr_15_Mn_15_, (**D**) Al_50_Ti_20_Cr_20_Mn_10_ and (**E**) Al_50_Ti_25_Cr_15_Mn_10_ alloys.

**Figure 2:**
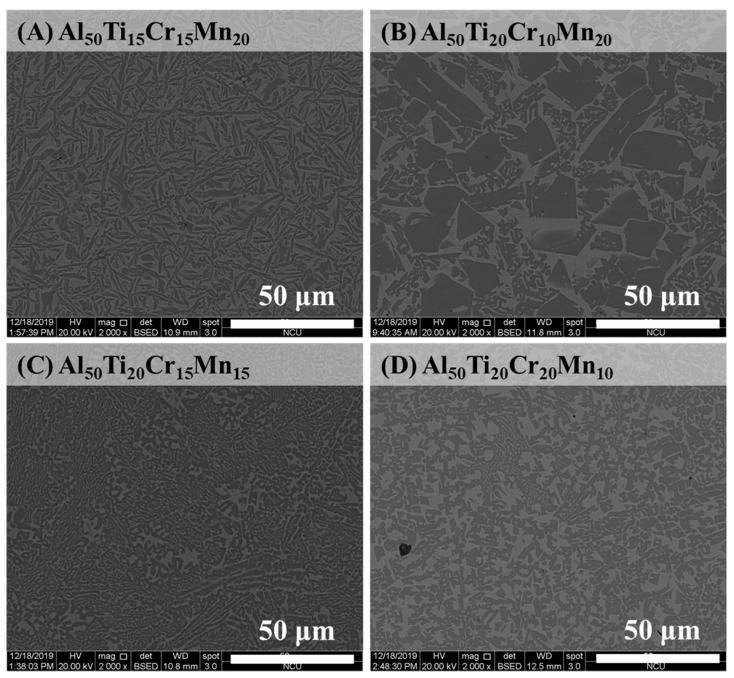
The SEM (scanning electron microscope) images of as-cast (**A**) Al_50_Ti_15_Cr_15_Mn_20_, (**B**) Al_50_Ti_20_Cr_10_Mn_20_, (**C**) Al_50_Ti_20_Cr_15_Mn_15_, and (**D**) Al_50_Ti_20_Cr_20_Mn_10_ alloys.

**Figure 3:**
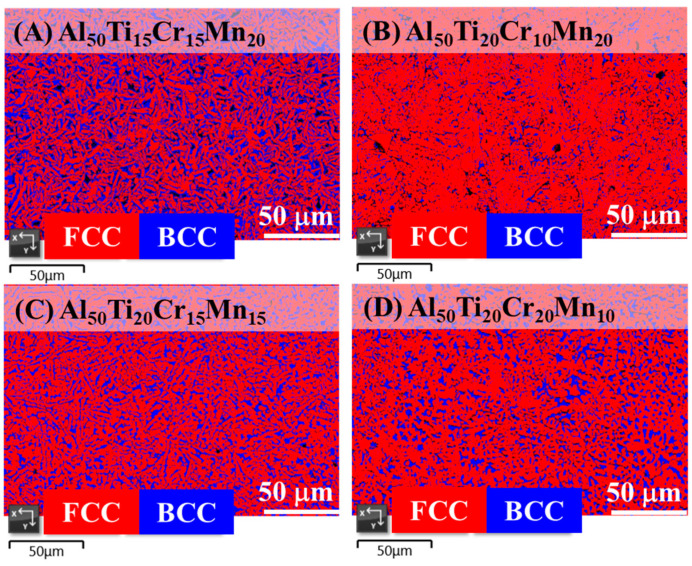
EBSD (electron backscatter diffraction) phase map indicating both face-centered cubic (FCC) and body-centered cubic (BCC) phases in the as-cast (**A**) Al_50_Ti_15_Cr_15_Mn_20_, (**B**) Al_50_Ti_20_Cr_10_Mn_20_, (**C**) Al_50_Ti_20_Cr_15_Mn_15_, and (**D**) Al_50_Ti_20_Cr_20_Mn_10_ alloys.

Figure 1A–D indicate that the microstructure of the as-cast Al-Ti-Cr-Mn alloys was composed of BCC and FCC phases. However, the as-cast Al_50_Ti_25_Cr_15_Mn_10_ alloy was not only composed of the BCC and FCC dual phase but also contained a few intermetallic phases, as illustrated in Figure 1E. The proportion of element Al to Ti was considerably high, facilitating the formation of intermetallic compounds due to the negative heat of mixing. Figure 2 indicates that the SEM images of the as-cast Al_50_Ti_20_Cr_20_Mn_10_, Al_50_Ti_20_Cr_15_Mn_15_, Al_50_Ti_20_Cr_10_Mn_20_, and Al_50_Ti_15_Cr_15_Mn_20_ alloys consisted of dark and bright areas. According to the peak intensity of XRD patterns and EBSD phase mapping analysis, as illustrated in Figure 3, the bright area in the SEM images was identified as the BCC phase and the dark area as the FCC phase. In addition, the results of electron microprobe (EPM) analysis for the as-cast Al_50_Ti_20_Cr_10_Mn_20_ alloy indicated that the bright area was enriched with Cr and Mn and dark areas were enriched with Ti and Al, as shown in Table 2.

By calculating the proportion of the dark and bright areas from SEM images, the fraction of the BCC phase of these alloys can be estimated, as presented in Table 3. On increasing Cr and Mn contents in the alloy, the proportion of the BCC phase (bright areas) increased slightly. By contrast, the proportion of the FCC phase increased with increasing Ti content. Therefore, based on the results of SEM images and EPM analysis, elements Cr and Mn were considered as the BCC stabilizers and Ti was considered as the FCC stabilizer in these dual-phase alloys. 

Table 3 lists the hardness results of alloys, which revealed that the hardness values of these dual-phase alloys were in the range of 300–400 Hv. However, all of the alloys exhibited similar hardness values for the as-cast and homogenized samples, except the Al_50_Ti_25_Cr_15_Mn_10_ alloy, which contained some intermetallic compounds.

**Figure 4:**
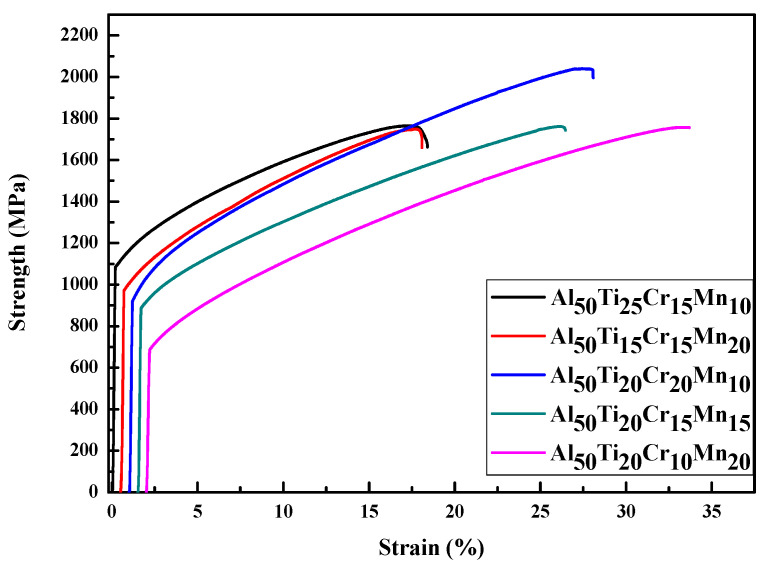
The mechanical compressive stress/strain curves for as-cast Al_50_Ti_25_Cr_15_Mn_10_, Al_50_Ti_15_Cr_15_Mn_20_, Al_50_Ti_20_Cr_20_Mn_10_, Al_50_Ti_20_Cr_15_Mn_15_ and Al_50_Ti_20_Cr_10_Mn_20_ alloys.

Figure 4 presents the results of compression tests for all Al-Ti-Cr-Mn system alloys. Among these alloys, the Al_50_Ti_20_Cr_10_Mn_20_ alloy contained 29.4 vol.% of the BCC phase and exhibited low compressive yield strength (648 MPa) and high ductility (32%). By contrast, the Al_50_Ti_15_Cr_15_Mn_20_ alloy contained 49 vol.% of the BCC phase and exhibited the highest compressive yield strength (944 MPa) and low ductility (17%). All compressive results are presented in Table 3. This finding confirmed that BCC-structured HEAs exhibit relatively high hardness and strength; however, they mostly exhibit brittleness [18].

The metallography of the as-cast Al_50_Ti_15_Cr_15_Mn_20_ alloy indicated that the flake-shaped FCC phase was surrounded by the BCC phase, as illustrated in Figure 2A. Flake-shaped phases adversely affect mechanical properties because the stress concentration at the edges of flake-shaped phases would cause the cracks to propagate rapidly [19,20]. On contrary, the Al_50_Ti_20_Cr_20_Mn_10_ alloy with the 35 vol.% BCC phase and a highly equi-axial shape FCC phase can yield high ductility due to the blunt edge of FCC phases.

**Figure 5:**
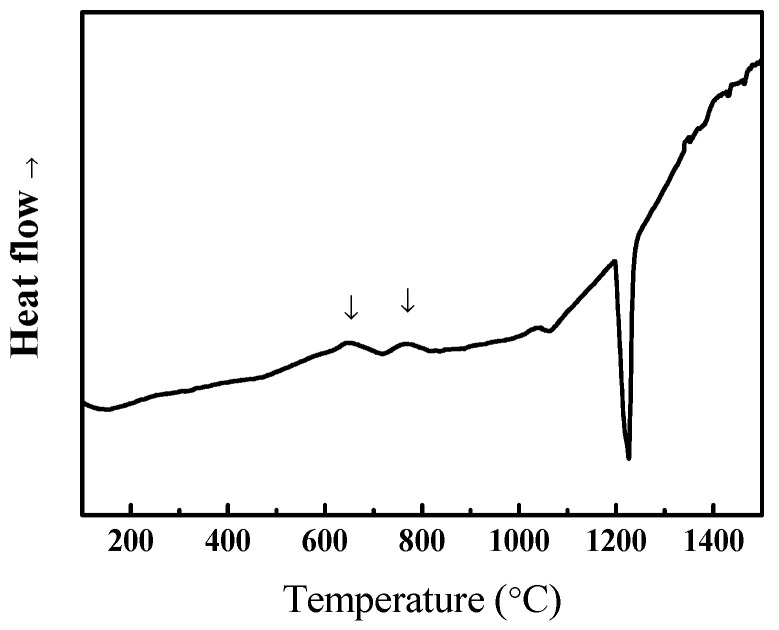
The DSC (differential scanning calorimeter) curves of the as-cast Al_50_Ti_20_Cr_10_Mn_20_ alloys.

**Figure 6:**
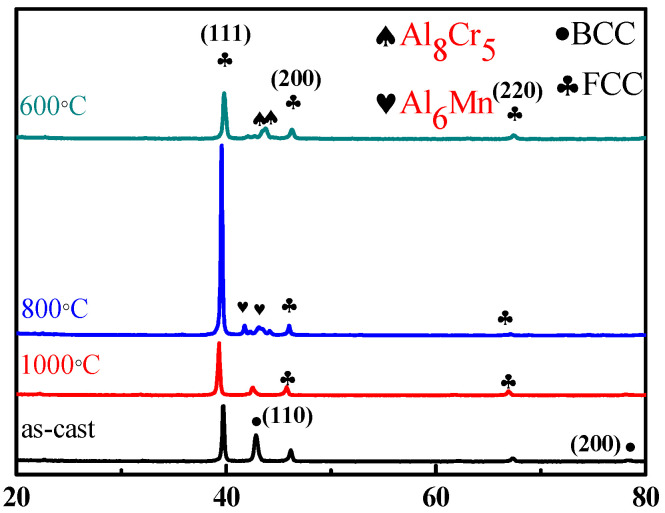
The XRD patterns of Al_50_Ti_20_Cr_10_Mn_20_ alloys in the as-cast condition and different heat treatment.

**Figure 7:**
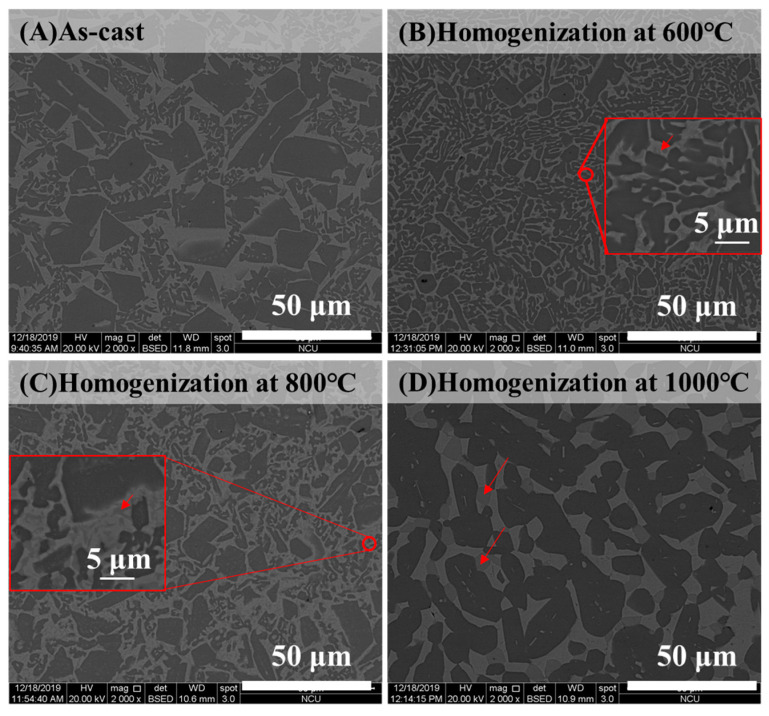
The SEM images of Al_50_Ti_20_Cr_10_Mn_20_ alloys in (**A**) as-cast, (**B**) homogenization at 600 °C, (**C**) homogenization at 800 °C, and (**D**) 1000 °C for 24 h.

**Figure 8:**
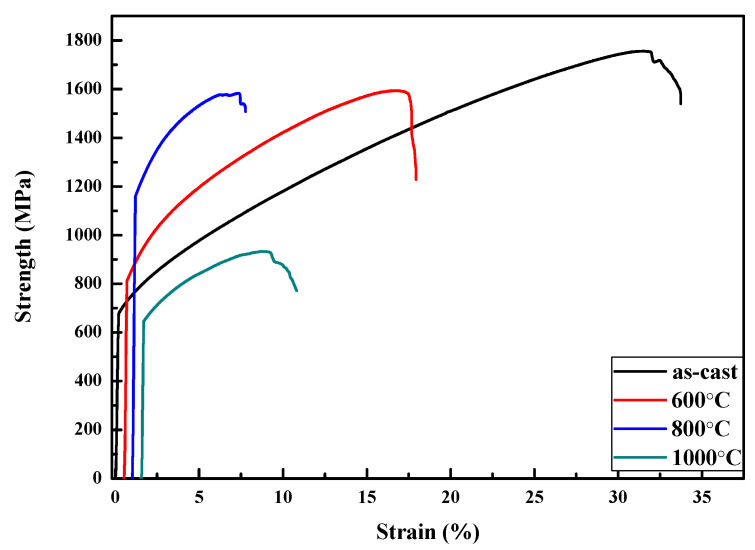
The mechanical compressive stress/strain curves for Al_50_Ti_20_Cr_10_Mn_20_ alloys in the as-cast condition and different heat treatment.

To further modify the morphology of BCC and FCC phases, the as-cast alloy with the highest ductility was selected as a representative alloy for homogenization at different high temperatures. Before heat treatment, the phase transformation analysis for the as-cast Al_50_Ti_20_Cr_10_Mn_20_ alloy was conducted using differential scanning calorimetry (DSC). The DSC curve indicated that two slightly exothermic precipitation reactions occurred between 600 and 800 °C, as illustrated in Figure 5. From the XRD results as illustrated in Figure 6, some intermetallic phases formed from the dual-phase matrix for the alloy heat treated at 600 and 800 °C, respectively. The minor intermetallic phases were also found between the BCC and FCC phases, as displayed in Figure 7B,C. It was noted that the SEM images of samples after 1000 °C, as shown in Figure 7D, revealed that the intermetallic phases could be obviously observed, although they were not detected by XRD and DSC analysis. Accompanied with the formation of intermetallic phases, the mechanical properties of heat-treated samples decreased visibly, for example, plastic strain decreased to <20%, as presented in Figure 8 and Table 4. Moreover, it was found that the effect of the BCC phase fraction still has a big role in the mechanical properties of the annealed samples.

### 3.3. Microstructure and Mechanical Properties of Quinary Al-Ti-Cr-Mn-V MEAs

**Figure 9:**
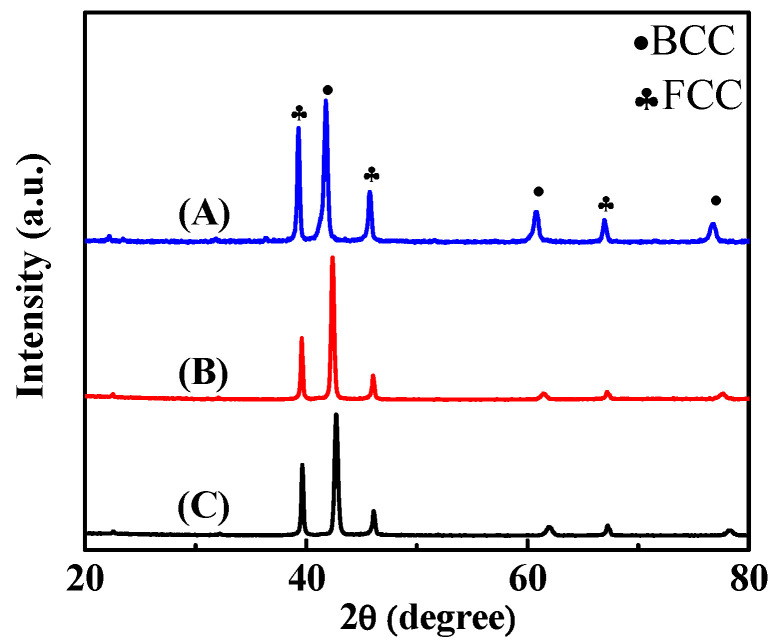
The XRD patterns of the as-cast (**A**) Al_50_(TiCrMn)_45_V_5_, (**B**) Al_50(_TiCrMn)_37.5_V_12.5_ and (**C**) Al_50_(TiCrMn)_30_V_20_ alloys.

**Figure 10:**
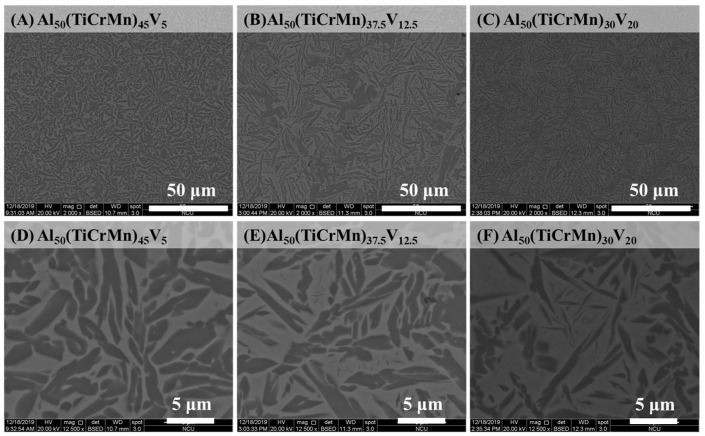
The SEM images of the as-cast (**A**) Al_50_(TiCrMn)_45_V_5_, (**B**) Al_50_(TiCrMn)_37.5_V_12.5_ and (**C**) Al_50_(TiCrMn)_30_V_20_ alloys, (**D**) Al_50_(TiCrMn)_45_V_5_, (**E**) Al_50_(TiCrMn)_37.5_V_12.5_, (**F**) Al_50_(TiCrMn)_30_V_20_.

**Figure 11:**
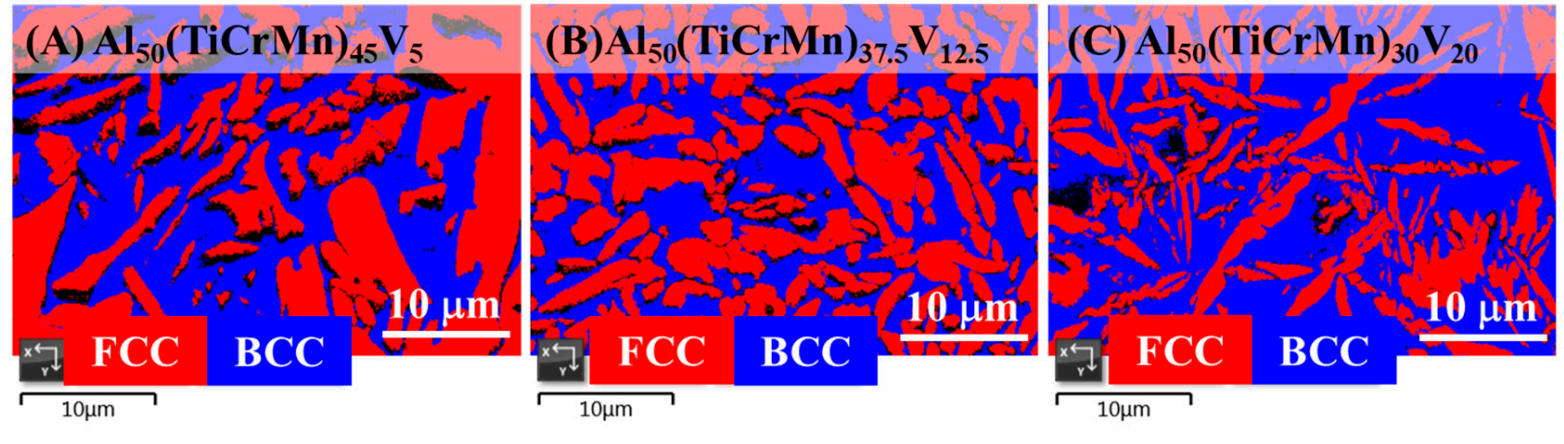
EBSD phase map indicating both FCC and BCC phases in the as-cast (**A**) Al_50_(TiCrMn)_45_V_5_, (**B**) Al_50_(TiCrMn)_37.5_V_12.5_ and (**C**) Al_50_(TiCrMn)_30_V_20_ alloys.

To further enhance the mechanical properties of the Al-Ti-Cr-Mn alloy, the alloy composition was fine-tuned by adding appropriate amounts of V, and the resulting alloys were named as Al_50_(TiCrMn)_45_V_5_, Al_50_(TiCrMn)_37.5_V_12.5_, and Al_50_(TiCrMn)_30_V_20_. The XRD patterns of all the as-cast Al_50_(TiCrMn)_45_V_5_, Al_50_(TiCrMn)_37.5_V_12.5_, and Al_50_(TiCrMn)_30_V_20_ alloys revealed a dual-phase structure, as illustrated in Figure 9. The SEM results also revealed that these Al-Ti-Cr-Mn-V alloys still consisted of BCC (bright areas) and FCC phases (dark areas), as illustrated in Figure 10. With the addition of V, the dimensions of FCC phases decreased considerably; the long-axial size became <1 μm, which was much smaller than that observed in quaternary Al-Ti-Cr-Mn alloys. In addition, the volume fraction of the BCC phase remarkably increased in quinary Al-Ti-Cr-Mn-V alloys and reached 61.1 vol.% of the BCC phase in Al_50_(TiCrMn)_30_V_20_ alloys, as presented in Figure 11.

**Figure 12:**
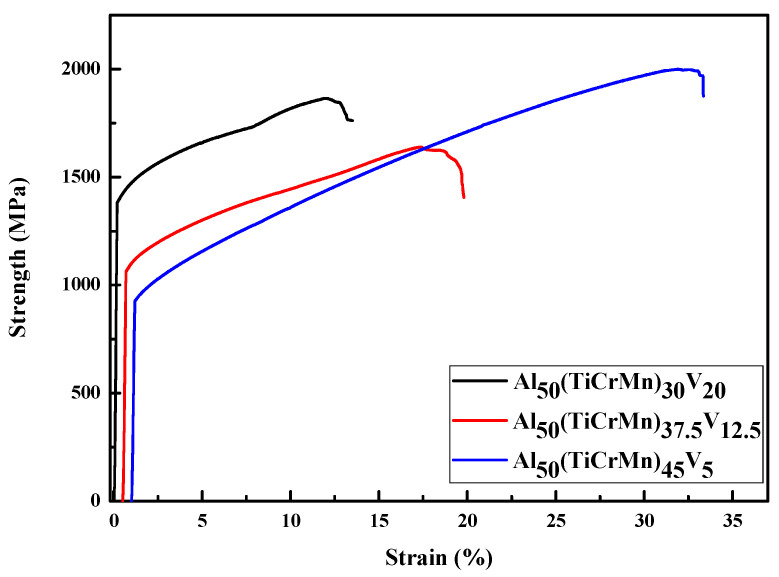
The mechanical compressive stress/strain curves for Al_50_(TiCrMn)_45_V_5_, Al_50_(TiCrMn)_37.5_V_12.5_ and Al_50_(TiCrMn)_30_V_20_ alloys.

As listed in Table 3, the hardness and mechanical properties of quinary Al-Ti-Cr-Mn-V alloys increased with the addition of V. In addition, the yield strength of Al-Ti-Cr-Mn-V alloys increased with increasing volume fraction of the BCC phase. The effect of a higher fraction of the BCC phase makes Al_50_(TiCrMn)_30_V_20_ alloys tougher (yield strength of 1360 MPa) but more brittle than Al_50_(TiCrMn)_45_V_5_ alloys. With a favorable combination of phase fractions and morphology, the Al_50_(TiCrMn)_45_V_5_ alloy exhibited the highest compressive strength (1995 MPa) and most prominent ductility (30%) among these three quinary alloys, as presented in Figure 12 and Table 3.

**Figure 13:**
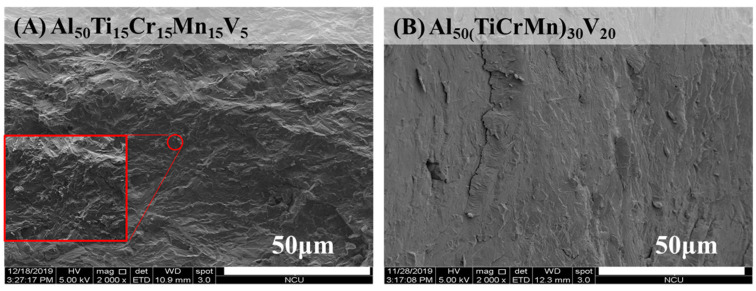
The SEM fractured images of as-cast (**A**) Al_50_Ti_15_Cr_15_Mn_15_V_5_ alloys and (**B**) Al_50_(TiCrMn)_30_V_20_ alloys.

Numerous rod-shaped FCC phases with blunt edges were surrounded by BCC phases in the Al_50_(TiCrMn)_45_V_5_ alloy. The combination of the fine dimensions of FCC and BCC phases and blunt edge of the FCC phase make the Al_50_(TiCrMn)_45_V_5_ alloy more ductile. However, the stress concentration caused by flake-shaped FCC phases with a sharp edge in the Al_50(_TiCrMn)_37.5_V_12.5_ and Al_50(_TiCrMn)_30_V_20_ alloys make these two alloys highly brittle. Therefore, the fracture surface of the Al_50_(TiCrMn)_45_V_5_ alloy exhibited denser dimples than that of the Al_50(_TiCrMn)_30_V_20_ alloy after compression tests, as presented in Figure 13A,B.

**Figure 14:**
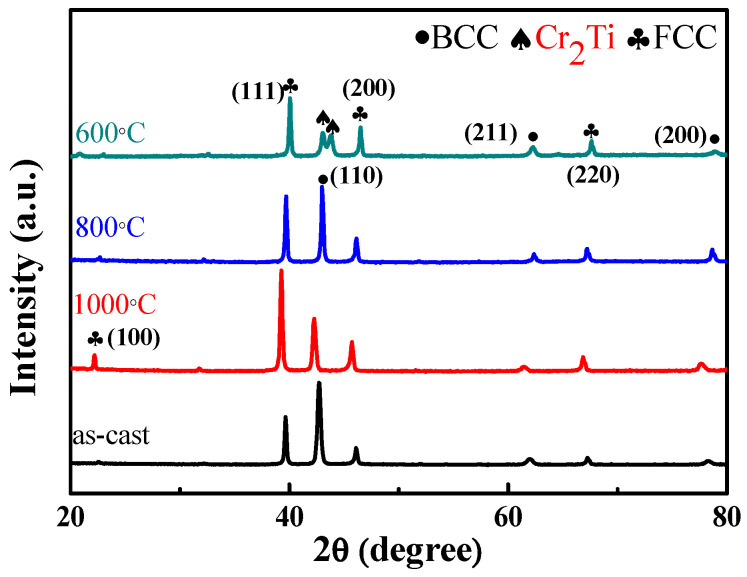
The XRD patterns of Al_50_Ti_15_Cr_15_Mn_15_V_5_ alloys in the as-cast condition and different heat treatments.

**Figure 15:**
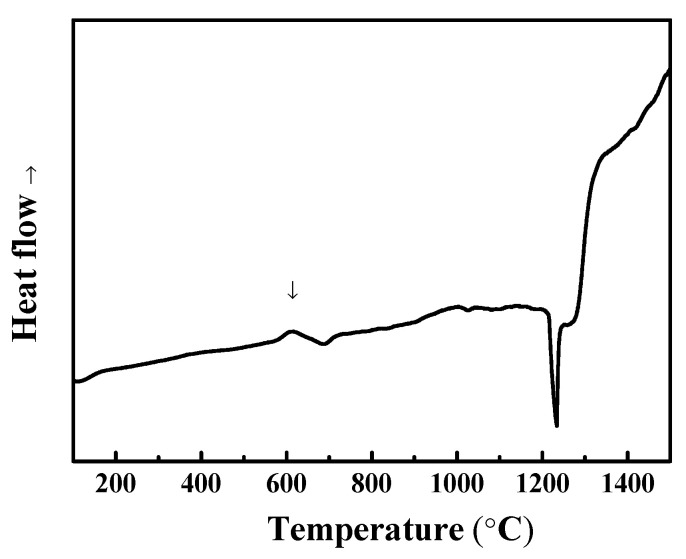
The DSC curves of the as-cast Al_50_Ti_15_Cr_15_Mn_15_V_5_ alloys.

**Figure 16:**
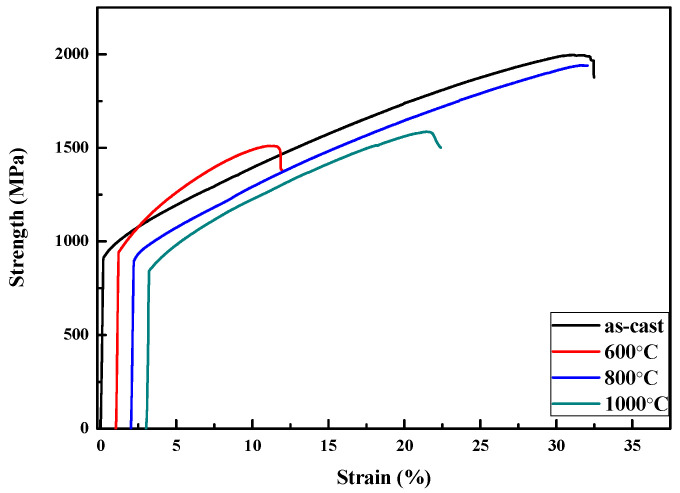
The mechanical compressive stress/strain curves for Al_50_Ti_15_Cr_15_Mn_15_V_5_ alloys in the as-cast condition and different heat treatments.

**Figure 17:**
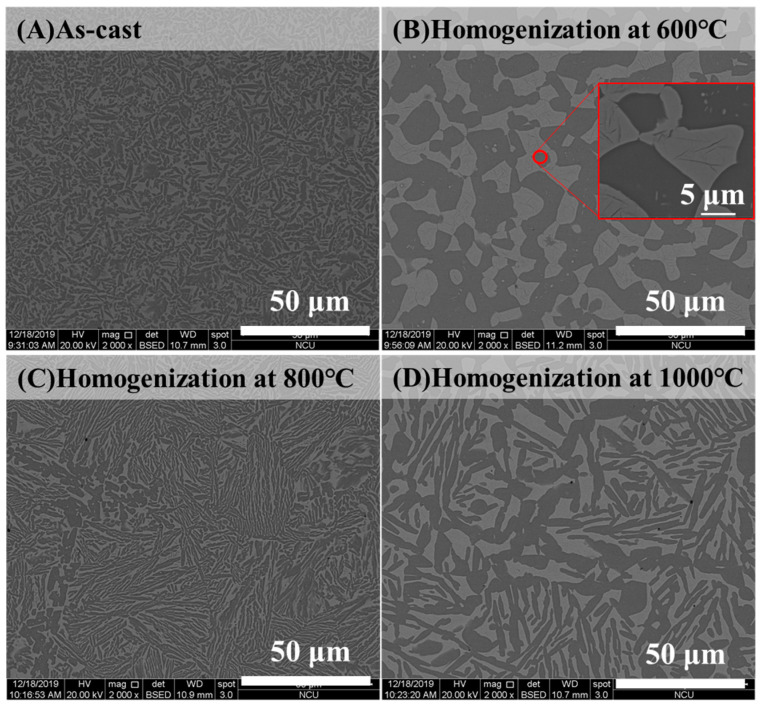
The SEM images of Al_50_Ti_15_Cr_15_Mn_15_V_5_ alloys in (**A**) as-cast, (**B**) homogenization at 600 °C, (**C**) homogenization at 800 °C, and (**D**) 1000 °C for 24 h.

Similarly, the Al_50_(TiCrMn)_45_V_5_ alloy was selected to further modify its morphology of dual-phase structures by homogenizing heat treatment at different temperatures, as illustrated in Figure 14. In Figure 15, the DSC curves indicate that precipitation occurred at approximately 600 °C, which proves that the microstructure of Al_50_(TiCrMn)_45_V_5_ alloys contained some intermetallic phases after heat treatment at 600 °C. In Figure 16, the results of the compressive test indicate that the Al_50_(TiCrMn)_45_V_5_ alloy exhibited a poor plasticity (<13% plastic strain) after 600 °C homogenizing. On the other hand, the Al_50_(TiCrMn)_45_V_5_ alloy still remains in the dual-phase structure after homogenization at 1000 °C, and even its ductility reduces to less than 20%, as shown in Table 4. As mentioned above, the effect of the BCC phase fraction is a main factor in the mechanical properties. After different heat treatments, the BCC phase faction of each annealed sample did not change significantly and it led to the similar yield strength of the alloys. In Figure 17A, it was observed that some needle-like intermetallic phases were precipitated in the BCC phase and it verified the poor mechanical properties of the annealed samples after heat treatment at 600 °C. The decrease in plastic strain of the alloy after 1000 °C homogenization is suggested to be due to the large dimension of the island-like FCC phase, which aggregated after high temperature homogenization, and meant that the BCC phases could not be covered with the numerous and thinner FCC phases, as shown in Figure 17D. The island-like FCC phase may cause stress concentration during the mechanical test and result in more brittle fracture behavior. Conversely, the morphology of the Al_50_(TiCrMn)_45_V_5_ alloy after 800 °C homogenization still maintained a similar morphology of the BCC and FCC phases as that of the as-cast alloy, as shown in Figure 17A,C. Accordingly, the Al_50_(TiCrMn)_45_V_5_ alloy has similar high mechanical properties to the as-cast sample and 800 °C homogenized sample.

## 4. Conclusions

The novel nonequiatomic lightweight Al-Ti-Cr-Mn and Al-Ti-Cr-Mn-V MEAs with dual-phase structures were successfully prepared using arc melting and drop casting and were explored. The results of microstructure analyses and mechanical property tests are summarized as follows:

1. All the designed alloys in this study achieved the density goal, which was <5.0 g/cm^3^.
2. The findings of the investigation of the quaternary Al-Ti-Cr-Mn alloy system confirmed that the Cr and Mn elements are considered BCC stabilizers, whereas Ti is considered the FCC stabilizer in these dual-phase alloys.
3. The mechanical properties of the quaternary Al_-_Ti-Cr-Mn alloy can be further improved by fine tuning its composition by adding V. The Al_50(_TiCrMn)_45_V_5_ alloy exhibits the highest compression strength (1995 ± 60 MPa) and ductility (33 ± 3%) among the designed quinary alloys.
4. With a low density (4.5 g/cm^3^), the Al_50_(TiCrMn)_45_V_5_ alloy exhibits a high specific compressive strength of 0.443 GPa·g/cm^3^ and excellent ductility. However, the Al_50_(TiCrMn)_45_V_5_ alloy maintains the original mechanical properties after homogenization for 24 h at 800 °C. These results are helpful for further designing the dual-phase HEAs/MEAs by using the nonequiatomic concept in the future.

## Figures and Tables

**Table 1:** The density of the Al-Ti-Cr-Mn and Al-Ti-Cr-Mn-V alloys.

	Theoretical Density (g/cm^3^)	Measured Density (g/cm^3^)
Al_50_Ti_20_Cr_20_Mn_10_	4.20	4.37
Al_50_Ti_20_Cr_15_Mn_15_	4.21	4.38
Al_50_Ti_20_Cr_10_Mn_20_	4.22	4.37
Al_50_Ti_25_Cr_15_Mn_10_	4.10	4.23
Al_50_Ti_15_Cr_15_Mn_20_	4.32	4.56
Al_50_(TiCrMn)_45_V_5_	4.27	4.50
Al_50_(TiCrMn)_37.5_V_12.5_	4.27	4.47
Al_50_(TiCrMn)_30_V_20_	4.27	4.40

**Table 2:** The EPM (electron microprobe) analysis of the as-cast Al_50_Ti_20_Cr_10_Mn_20_ alloys.

	Al	Ti	Cr	Mn
Nominal composition (at.%)	50.00	20.00	10.00	20.00
Bright phase (BCC)	36.27	9.78	26.86	27.09
Dark phase (FCC)	57.96	24.07	4.42	13.54

**Table 3:** The mechanical hardness and compressive properties of the Al-Ti-Cr-Mn and Al-Ti-Cr-Mn-V alloys.

Constituent	BCC Fraction (%)	Hardness (Hv)	Yield Strength (MPa)	Ultimate Strength (MPa)	Ductility (%)
Al_50_Ti_15_Cr_15_Mn_20_	49.4	395 ± 4	944 ± 28	1749 ± 52	17 ± 2
Al_50_Ti_20_Cr_20_Mn_10_	34.8	347 ± 6	855 ± 26	2043 ± 61	27 ± 3
Al_50_Ti_20_Cr_15_Mn_15_	32.1	346 ± 5	780 ± 23	1772 ± 53	25 ± 2
Al_50_Ti_20_Cr_10_Mn_20_	29.4	335 ± 8	648 ± 19	1763 ± 53	32 ± 2
Al_50_Ti_25_Cr_15_Mn_10_	24.8	406 ± 6	1128 ± 34	1740 ± 52	17 ± 2
Al_50_(TiCrMn)_45_V_5_	40.0	355 ± 8	934 ± 28	1995 ± 60	33 ± 3
Al_50_(TiCrMn)_37.5_V_12.5_	54.8	385 ± 1	1059 ± 32	1602 ± 48	18 ± 2
Al_50_(TiCrMn)_30_V_20_	61.1	489 ± 13	1362 ± 41	1823 ± 55	12 ± 1

**Table 4:** The mechanical compressive properties of the Al-Ti-Cr-Mn and Al-Ti-Cr-Mn-V alloys in the as-cast condition and different heat treatment.

Constituent	BCC Fraction (%)	Yield Strength (MPa)	Ultimate Strength (MPa)	Ductility (%)
Al_50_Ti_20_Cr_10_Mn_20_	29.5	648 ± 19	1763 ± 53	32 ± 2
Al_50_Ti_20_Cr_10_Mn_20_ (600 °C)	31.1	822 ± 24	1598 ± 48	17 ± 2
Al_50_Ti_20_Cr_10_Mn_20_ (800 °C)	34.3	1161 ± 35	1583 ± 47	6 ± 1
Al_50_Ti_20_Cr_10_Mn_20_ (1000 °C)	27.7	650 ± 19	941 ± 28	8 ± 1
Al_50_(TiCrMn)_45_V_5_	40.5	934 ± 28	1995 ± 60	33 ± 3
Al_50_(TiCrMn)_45_V_5_ (600 °C)	37.5	950 ± 28	1514 ± 45	10 ± 1
Al_50_(TiCrMn)_45_V_5_ (800 °C)	37.7	900 ± 27	1946 ± 58	30 ± 2
Al_50_(TiCrMn)_45_V_5_ (1000 °C)	37.1	846 ± 25	1588 ± 47	19 ± 2
